# A Case Report: Kikuchi Disease Associated With a Positive Auto-Immune Panel Triggered by COVID-19 Infection

**DOI:** 10.7759/cureus.50911

**Published:** 2023-12-21

**Authors:** Andrew Graef, Aimee Willett, Andrew-Huy Dang, Jayalakshmi Balakrishna, Charles Nicely, Robert Baiocchi

**Affiliations:** 1 Internal Medicine, OhioHealth Riverside Methodist Hospital, Columbus, USA; 2 Internal Medicine, The Ohio State University Wexner Medical Center, Columbus, USA; 3 Hematopathology, The Ohio State University Wexner Medical Center, Columbus, USA; 4 Hematopathology, OhioHealth Riverside Methodist Hospital, Columbus, USA; 5 Hematology and Oncology, The Ohio State University Wexner Medical Center, Columbus, USA

**Keywords:** major hyperferritinemia, covid-19, necrotizing histiocytic lymphadenitis, s: kikuchi-fujimoto disease, kikuchi disease

## Abstract

Kikuchi disease (KD) is a rare, benign inflammatory condition characterized by fever and cervical lymphadenopathy. While the pathogenesis is largely unknown, Kikuchi disease onset has strong associations with various infections and autoimmune conditions. There are few reported cases of Kikuchi disease triggered by coronavirus disease 2019 (COVID-19) infection or vaccination.

A 43-year-old Filipina female with a history of anemia and recent uncomplicated COVID-19 infection one month prior presented with a one-month history of progressive weakness, fatigue, anorexia with 30-pound weight loss, fevers, odynophagia, and new-onset hematemesis. Initial laboratory findings were most significant for a markedly elevated ferritin level prompting initial concern for hemophagocytic lymphohistiocytosis. Admission imaging revealed diffuse cervical and thoracic lymphadenopathy. Lymph node biopsy revealed paracortical expansion with numerous histiocytes with phagocytosed necrotic debris and germinal center necrosis, consistent with Kikuchi disease. She received supportive care without any medical intervention and improved clinically with the resolution of lymphadenopathy and inflammatory laboratory markers.

This report describes the initial presentation and subsequent diagnostic workup of a unique and infrequently documented case of Kikuchi disease secondary to COVID-19 infection. This case highlights general constitutional symptoms, including fever and lymphadenopathy as defining characteristics of Kikuchi disease. During diagnostic workup, it is important to rule out hematologic emergencies, such as hemophagocytic lymphohistiocytosis, which can present similarly. This case also reports a concurrent autoimmune workup, which was positive at the time of the Kikuchi disease diagnosis. COVID-19 infections and deaths, while declining in the post-pandemic period, remain significant, thus diagnostic consideration for conditions of self-limited disorders, such as Kikuchi disease, should be considered.

## Introduction

Kikuchi disease (KD), also known as histiocytic necrotizing lymphadenitis or Kikuchi-Fujimoto disease after the Japanese pathologist who first described it in 1972 [1], is an inflammatory condition characterized by cervical lymphadenopathy and fever [2]. While KD is rare, it is fortunately benign and self-limiting [2]. Initial records indicate strong epidemiologic preferences for KD in young Asian females, however, more recent data show occurrences in increasingly diverse populations [3]. The pathophysiology of KD is largely unknown, with leading hypotheses encompassing both infectious and autoimmune triggers of an abnormal inflammatory response [3]. KD typically presents with acute, tender, and mobile cervical lymphadenopathy with associated fevers, rash, arthralgias, fatigue, and hepatosplenomegaly [3,4]. Laboratory studies can be significant for cytopenia. KD is diagnosed via lymph node excision with pathologic examination where histology reveals paracortical foci with necrosis and histiocytic cellular infiltrate without the presence of neutrophils or eosinophils. KD typically has a good prognosis; however, it can rarely be complicated by hemophagocytic lymphohistiocytosis (HLH) [5]. Management includes symptomatic control of fever and pain and monitoring for resolution, typically occurring within one to four months. The lifetime recurrence rate of KD is 3-4% [2].

In the era of the COVID-19 pandemic, a few recent cases of coronavirus disease 2019 (COVID-19) infection and severe acute respiratory syndrome coronavirus 2 (SARS-CoV-2) vaccination-associated KD have been reported [6-14]. Literature indicates KD occurrences following both COVID-19 vaccination and viral infection. The onset of KD following vaccine or virus exposure ranges between 10 and 90 days [10,14]. Our case highlights the importance of considering a thorough diagnostic workup prior to treatment initiation, as pursuing treatment for presumed HLH in an otherwise stable patient could lead to unnecessary morbidity and poor outcomes.

## Case presentation

**Figure 1:**
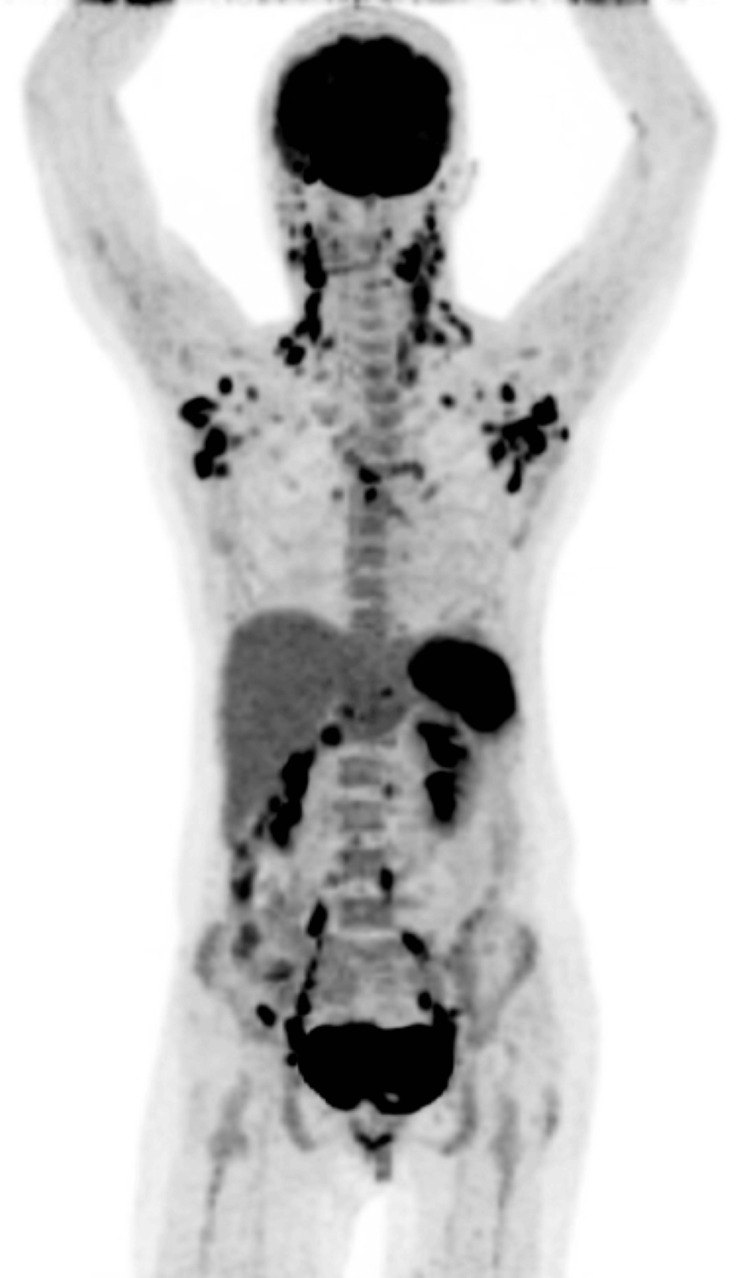
PET-CT scan illustrating hypermetabolic foci involving lymph nodes, spleen, and diffuse hypermetabolic bone marrow PET-CT: positron emission tomography-computed tomography

**Figure 2:**
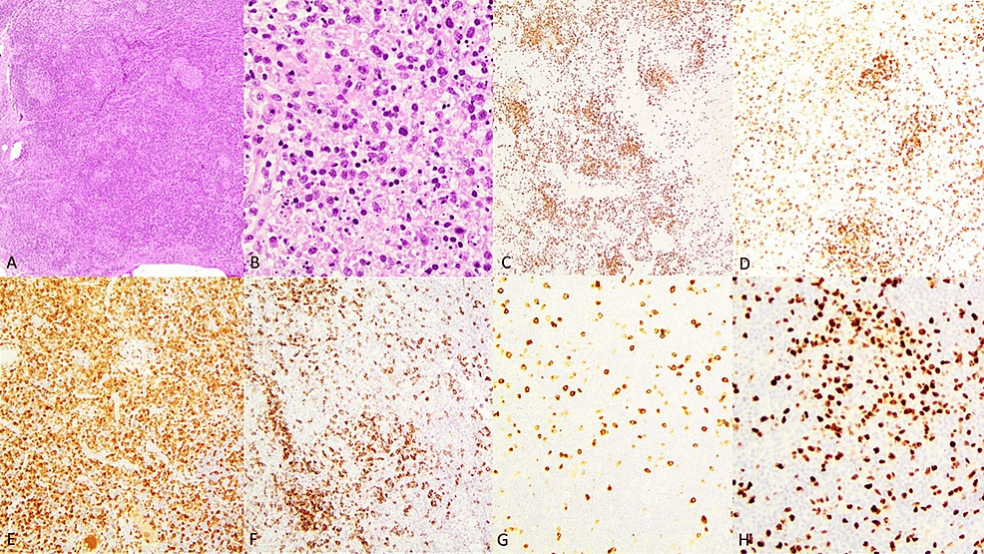
Left axillary lymph node biopsy A. Paracortical expansion and widely spaced follicles of varying sizes, HE, 4x. B. Paracortex containing mixed inflammatory cells with predominantly histiocytes and abundant karyorrhectic debris, HE, 40x. C. CD20 stain highlighting the regressed follicles and increased interfollicular B cells, CD20 4x. D. CD5 stain highlights the T cells in the paracortex, CD5 10x. E. CD163 stain shows numerous histiocytes, CD163 10x. F. CD138 shows increased plasma cells, 10x. G. CD30 highlights many immunoblasts, CD30, 10x. H. Ki67 shows increased proliferation in the interfollicular areas, Ki67, 20x.

A 43-year-old Filipina female with a past medical history of anemia presented with one month of progressive weakness, fatigue, anorexia, 30-pound weight loss, fevers, odynophagia, and one episode of hematemesis. One month prior, she was diagnosed with a COVID-19 infection (GeneXpert reverse transcription-polymerase chain reaction (RT-PCR)), which was managed with supportive care and resolved uneventfully. Physical exam was notable for a fever of 101.8 degrees Fahrenheit and cervical lymphadenopathy; no hepatosplenomegaly was appreciated. Labs on admission showed hemoglobin 7.5 g/dL, platelets 97 k/uL, ferritin 58,912 ng/mL, and iron saturation of 53%. Further workup revealed 1-2 schistocytes/hpf on the blood smear, lactate dehydrogenase (LDH) 1930 U/L, and undetectable haptoglobin. Human immunodeficiency virus and hepatitis C virus testing were negative, and hepatitis B virus testing showed immunity. Direct antiglobulin testing was negative. Additional labs revealed creatinine within normal limits; erythrocyte sedimentation rate 81 mm/hr, and triglycerides 316 mg/dL. Autoimmune workup was notable for an initially negative antinuclear antibody (ANA), however, repeat testing revealed a positive antinuclear antibody (ANA) as well as positive double-stranded DNA, anti-Smith, anti-ribonucleoprotein (RNP), and anti-SSA antibodies. Table 1 shows the lab values. Computed tomography (CT) imaging showed scattered subcentimeter pulmonary nodules, bilateral axillary lymphadenopathy, mild retroperitoneal adenopathy, and no hepatosplenomegaly. Further workup for possible underlying hemophagocytic lymphohistiocytosis (HLH) versus lymphoproliferative disorder revealed a soluble interleukin 2 (IL-2) receptor (sIL-2R) level of 2939 pg/ml (normal range 175-858) and IL-2 of 33.1 pg/ml (< 2). Bone marrow biopsy showed normocellular bone marrow with rare histiocytes with erythrophagocytosis. Acid-fast bacilli (AFB) and Grocott methenamine silver (GMS) staining were negative, and two separate interferon-gamma release assays were indeterminate. Subsequent testing for indolent infections, including fungal serologies, Mycobacterium tuberculosis (MTB)-PCR, and two sputum acid-fast bacilli (AFB) smears, was negative. Repeat COVID-19 infection during this evaluation was negative, which occurred 27 days after initial positive testing. The positron emission tomography (PET) scan showed diffuse hypermetabolic lymphadenopathy (SUV 8.9 - 12 range) with increased splenic and bone marrow uptake concerning for malignancy (Figure 1). Lymph node biopsy revealed paracortical expansion with mixed inflammatory cells, numerous histiocytes with phagocytosed cellular debris and red blood cells, abundant karyorrhectic debris, and some germinal centers with necrosis (Figure 2), suggestive of viral lymphadenitis or Kikuchi disease. Flow cytometry and PCR for B-cell and T-cell clonality did not identify any clonal B or T-cell populations. The patient received supportive treatment and close inpatient monitoring of clinical and laboratory results. Repeat ferritin, sIL-2R, ESR, LDH, triglyceride, and cytopenias downtrended. She was discharged on hospital day 16 with outpatient follow-up. Subsequent follow-up four months later showed continued symptomatic improvement with ferritin decreased < 500, LDH 170 U/L, and resolution of cytopenias. Immunology exome next-generation sequencing results failed to show any mutational variants associated with autoinflammatory conditions.

**Table 1:** Laboratory values and reference ranges LDH: lactate dehydrogenase; CMV: Cytomegalovirus; PCR: polymerase chain reaction; EBV: Epstein-Barr virus; MTB: Mycobacterium tuberculosis

	Admission Value	Follow-Up Value	Reference Range
Complete Blood Count			
Hemoglobin	7.5 g/dL	9.5 g/dL	12.0-16.0 g/dL
Platelets	108 k/mcL	488 k/mcL	150-400 K/mcL
Metabolic Panel			
Creatinine	0.42 mg/dL	0.55 mg/dL	0.40-1.10 mg/dL
Iron Studies			
Ferritin	58,912 ng/mL	470 ng/mL	10-291 ng/mL
Iron Saturation	53%	14%	20-50%
Hematology			
Schistocytes	1-2/hpf	NA	0/hpf
LDH	1,930 U/L	173 U/L	100-250 U/L
Haptoglobin	<10.0 mg/dL	NA	33.0-171.0 mg/dL
Direct Coombs Test	Negative	NA	Negative
Infectious Disease			
HIV Antibody (HIV1/HIV2)	Negative	NA	Negative
Hepatitis C Antibody	Negative	NA	Negative
Hepatitis B Surface Antibody	Positive	NA	Negative
Hepatitis B Surface Antigen	Negative	NA	Negative
Hepatitis B Core Antibody	Negative	NA	Negative
CMV PCR	<50 IU/mL	NA	<50 IU/mL
EBV PCR	<1,000 IU/mL	NA	<1,000 IU/mL
Aspergillus Antibody	<1:8	NA	<1:8
Coccidiosis Antibody	<1:2	NA	<1:2
Blastomyces Antibody	0.5	NA	<0.91
Histoplasma mycelial Antibody	<1:8	NA	<1:8
Histoplasma Yeast Antibody	<1:8	NA	<1:8
MTB-PCR	Negative	NA	Negative
Acid-Fast Bacilli, Sputum	Negative x2	NA	Negative
Lipid Panel			
Triglycerides	316 mg/dL	148 mg/dL	30-150 mg/dL
Autoimmune			
Antinuclear Antibody (ANA)	<1:80	NA	<1:80
Double-Stranded DNA (ds-DNA)	16 IU/mL	NA	<4 IU/mL
Anti-Smith	Positive	NA	Negative
Anti-Ribonucleoprotein (RNP)	Positive	NA	Negative
Anti-SSA	Positive	NA	Negative
Immunology			
Erythrocyte Sedimentation Rate	81 mm/hr	57 mm/hr	0-20 mm/hr
Soluble Interleukin-2 Receptor (sIL-2R)	2,939 pg/mL	1034 pg/mL	175.3-858.2 pg/mL

## Discussion

This case illustrates an infrequent but documented association between recent COVID-19 infection and KD. Our patient presented with signs and symptoms of cytokine storm, not dissimilar from many of the critical presentations of severe active COVID-19 disease. Her workup was immediately concerning for an autoinflammatory hematologic emergency, such as HLH or macrophage activation syndrome (MAS), seen in the setting of COVID-19 disease. Also high on the differential diagnosis was a lymphoproliferative disorder, with an aggressive B-cell lymphoma being of high concern. Interestingly, this patient met the clinical criteria for HLH/MAS, with five of the necessary criteria, including fever, bicytopenia, fasting hypertriglyceridemia, evidence of erythrophagocytosis on bone marrow biopsy, and an elevated soluble IL-2 receptor antibody. MAS shares many overlapping features with HLH, although it is more commonly associated with autoimmune diseases than viral infections. This patient’s positive autoimmune serology made this case more in line with MAS. This illustrates the importance of a histologic diagnosis, as the subsequent treatment pathway is markedly different when considering each diagnosis (lymphoma vs. benign condition like KD vs MAS) [4]. Immunosuppression or chemotherapy would be warranted in HLH/MAS and lymphoma, respectively, while Kikuchi disease is known to be a self-limited condition that will regress without treatment in most cases, although in aggressive or recurrent cases steroids may be utilized [4,9,12]. This patient’s lymph node biopsy results and spontaneous clinical improvement were consistent with the clinical course of Kikuchi disease. Without intervention, all concerns for uncontrolled autoinflammation also resolved.

This patient’s workup is also unique in that she had numerous positive auto-antibodies that could be consistent with systemic lupus erythematosus (SLE) or another mixed connective tissue disease. Thus far, no previous COVID-19 and Kikuchi disease cases have been noted to have a positive autoimmune panel other than singularly positive ANA [12]. Kikuchi disease has been reported in association with SLE in about 3% of cases [15], with other rare associations with Sjogren’s syndrome [16]. Stimson et al. reported a case of a 17-year-old male with several weeks of B symptoms and lymphadenopathy in the setting of a recent COVID-19 infection [17]. He was also noted to have a bicytopenia but had a negative ANA screen. While the significance of the autoimmune results in our patient is still unclear, her clinical and laboratory improvement in the absence of systemic treatment seems to argue against a concomitant new diagnosis of SLE. While she met numerous European Alliance of Associations for Rheumatology (EULAR) criteria for a diagnosis of SLE, all of the features were deemed more likely to have an alternative cause.

## Conclusions

This case illustrates the complex array of manifestations that can occur following infection with Sars-CoV-2 and COVID-19 illness. While declining, COVID-19 illness and related morbidity and mortality remain a global health problem, especially in regions with low vaccination rates. Consideration of a broad differential diagnosis, including malignant and self-limiting conditions, in such complex patients is essential.
